# Renal Failure among Women of Reproductive Age in Burundi: Estimating the Prevalence and Associated Factors Using Population-Based Data

**DOI:** 10.1155/2021/6640495

**Published:** 2021-02-27

**Authors:** Michael Ekholuenetale, Temitope Oluwaseyi Adeyoju, Herbert Onuoha, Amadou Barrow

**Affiliations:** ^1^Department of Epidemiology and Medical Statistics, Faculty of Public Health, College of Medicine, University of Ibadan, Ibadan, Nigeria; ^2^Department of Demography and Population Studies, School of Social Sciences, University of Witwatersrand, Johannesburg, South Africa; ^3^Department of Tropical Hygiene and Public Health, Medical Faculty, Heidelberg University, Heidelberg, Germany; ^4^Department of Public & Environmental Health, School of Medicine & Allied Health Sciences, University of The Gambia, Kanifing, Gambia

## Abstract

**Background:**

Renal failure is a leading cause of morbidity and mortality in many resource-constrained settings. In developing countries, little has been known about the prevalence and predisposing factors of renal failure using population-based data. The objective of this study was to examine the prevalence and associated factors of renal failure among women of reproductive age in Burundi.

**Methods:**

We used nationally representative cross-sectional data from the 2016-2017 Burundi Demographic and Health Survey (BDHS). Data on 17,269 women of reproductive age were included. The outcome variable was a renal failure as determined by the patient's report. Percentage, chi-square test, and multivariable logistic regression model were used to analyze the data. The results from the logistic regression model were presented as adjusted odds ratio (AOR) and confidence interval (95% CI). The significance level was set at *p* < 0.05.

**Results:**

The overall prevalence of renal failure was 5.0% (95% CI: 4.4%, 5.7%). Higher-aged women were more likely to have a renal failure when compared with women aged 15–19 years. Rural dwellers were 1.65 times as likely to have a renal failure when compared with women in the urban residence (AOR = 1.65; 95% CI: 1.24, 2.20). Women who had secondary + education had a 39% reduction in the odds of renal failure when compared with women with no formal education (AOR = 0.61; 95% CI: 0.46, 0.81). Health insurance coverage accounted for a 23% reduction in the odds of renal failure when compared with women who were not covered by health insurance (AOR = 0.77; 95% CI: 0.63, 0.93). Women who had a terminated pregnancy were 1.50 times as likely to have a renal failure when compared with women with no history of terminated pregnancy (AOR = 1.50; 95% CI: 1.24, 1.82). Furthermore, women with a history of contraceptive use were 1.32 times as likely to have a renal failure when compared with women without a history of contraceptive use (AOR = 1.32; 95% CI: 1.11, 1.57).

**Conclusion:**

Lack of formal education, having no health insurance coverage, and ever used anything or tried to delay or avoid getting pregnant were the modifiable risk factors of renal failure. The nonmodifiable risk factors were old age, rural residence, certain geographical regions, and having a history of pregnancy termination. Understanding the risk factors of renal failure will help to instigate early screening, detection, and prompt treatment initiation. In addition, early detection of the risk factors can help to reduce the adverse health impact including maternal death.

## 1. Background

Globally, renal failure affects approximately 10% of the world's adult population [1]. The global number of people with renal failure reached 752.7 million in 2016, with a disproportionate 417.0 million women [2]. Albuminuria with retained glomerular filtration rate was the most prevalent type of impaired renal function, estimated to affect 260.1 million women worldwide (62.4 percent from renal failure in women) [2]. With women having an unfair share of renal failure, it becomes very important to investigate the etiology of the disease among this key population. Renal failure is a major risk factor for premature death and cardiovascular disease [3]. Renal failure affects people of all classes, ages, nationalities, and geographical settings [4]. Impaired kidney function could lead to a great share of disability and death. Among the individuals with renal failure, the stage is classified by the level of glomerular filtration rate, with higher stages representing lower glomerular filtration rate levels [5, 6].

African descendants have been reported to have a high risk of renal failure occurrence and progression to end-stage renal disease [7]. No doubt, several African countries are undergoing rapid epidemiological transitions and are faced with the double burden of diseases, somewhat driven by changes in lifestyles and environmental changes such as rapid urbanization [8]. Consequently, this has led to a rise in the sufferers of renal failure in African [9]. Due to the increased risk factors [10], it is widely seen as a key public health problem, against the background of unavailability and inaccessibility to renal replacement therapy in many African settings [9]. This presupposes, therefore, that prevention and early detection of renal failure are essential as mitigation strategies. Another notable challenge is the lack of accurate data on renal failure exact magnitude in the continent, despite the fact that the number of reports on prevalence across Africa has increased in recent years [9]. A systematic review of renal failure prevalence in Africa was limited to sub-Saharan Africa countries and specifically noted the inability to make definitive inferences due to the poor quality of included studies [11].

Several nonmodifiable risk factors have been identified to be associated with renal failure including older age, race, acute kidney injury, metabolic syndrome, a history of cardiovascular disease, malignancy, hyperlipidemia, gender, hypertension, genetic component, family history and ethnicity, diabetes mellitus, and nephrotoxins, as well as modifiable risk factors, such as exposure to heavy metals, obesity, smoking, income, occupation, wealth, household situation, HIV infection, hepatitis C virus, socioeconomic status, low birth weight, excessive alcohol consumption, and use of analgesic medications [12–14]. There are burdens that renal failure brings on the victims, their families, and even the nation especially if renal failure is more prevalent among economically active persons, which affects any nation's productivity and economics. The economic burden can be mitigated by early detection of the risk factors [15]. It is worrisome that treatment of renal failure is hardly affordable where available. For example, continuous ambulatory peritoneal dialysis is an expensive treatment modality in Africa and raises complications as patients would have to miss several medical appointments and consequently die due to renal failure [16]. Some countries do not have the facilities to accommodate renal failure victims as it requires dialysis machines and expert doctors known as a nephrologist.

Reliable estimates of the global burden of renal failure using large population-based data have been conducted over time and in different geographical settings [1, 4, 17, 18]. A major challenge is that these burdens may vary by a certain spectrum of life: from poverty to affluence, from malnutrition to obesity, in agrarian to postindustrial settings, and along the life course from newborns to older people [19]. Again, several diseases result in renal complications, and many people who have renal failure lack access to care in poor-resource settings. The causes, consequences, and costs of renal failure have implications for public health policy in Burundi. Increasing economic and utilization of healthcare disparities, epidemiological transition, migration, unsafe working conditions and environmental hazards, natural disasters, and pollution may hinder attempts to reduce the morbidity and mortality from renal failure [11, 12, 20, 21]. The sustainable development goals (SDGs) emphasize the importance of mapping the actions toward achieving all of the targets that have the potential to improve understanding, measurement, prevention, and control of diseases [22, 23]. Ensuring healthy lives and promoting well-being for all ages is essential to sustainable development. In light of the above, we aimed to examine the prevalence and associated factors of renal failure among women of reproductive age in Burundi.

## 2. Methods

### 2.1. Data Sources

We used cross-sectional nationally representative data extracted from the 2016-17 Burundi Demographic and Health Survey (BDHS). A sample of 17,269 women aged 15–49 years was included in this study. BDHS data was collected through a stratified multistage cluster sampling technique. The procedure for the stratification approach divides the population into groups by geographical region and crossed by place of residence–urban versus rural. A multilevel stratification approach is used to divide the population into first-level strata and to subdivide the first-level strata into second-level strata, and so forth. A major advantage is that the sampling design and data collection approach are similar across countries which makes the results of different settings comparable. Though from the onset, DHS was designed to expand on fertility, demographic, and family planning data collected in the World Fertility Surveys and Contraceptive Prevalence Surveys, it has become the prominent source of population surveillance for the monitoring of population health indices, particularly in resource-constrained settings. BDHS has great merits with national coverage of high-quality data to enhance the understanding of epidemiological research that estimates prevalence, trends, and inequalities and by communicating them to policymakers. BDHS data is available in the public domain and accessed at http://dhsprogram.com/data/available-datasets.cfm.

### 2.2. Operational Definition of Variables

#### 2.2.1. Outcome Variable

The main outcome variable in this study was a renal failure as determined by the respondent's report. To derive this variable, BDHS asked the question: “*Suffering from renal failure?*” The respondents answered yes versus no. This was self-reported by the women based on their health condition.

#### 2.2.2. Explanatory Variables

The independent variables include women's age, residential status, geographical region, education, religion, exposure to media, wealth quintiles, marital status, health insurance coverage, participation in the labor force, parity, source of drinking water, sanitation, ever had a terminated pregnancy, body mass index, ever used anything or tried to delay or avoid getting pregnant, anemia status, smoking/use tobacco product, and alcohol use. These variables were categorized as follows: women's age: 15–19, 20–24, 25–29, 30–34, 35–39, 40–44, and 45–49; residential status: urban versus rural; geographical region: Bubanza, Bujumbura Rural, Bururi, Cankuzo, Cibitoke, Gitega, Karusi, Kayanza, Kirundo, Makamba, Muramvya, Muyinga, Mwaro, Ngozi, Rutana, Ruyigi, Bujumbura Mairie, and Rumonge; education: no formal education, primary, and secondary/higher; religion: Christianity, Islam, and traditional/no religion; exposure to media: yes versus no; marital status: unmarried, currently married/living with a partner, and formerly married; health insurance coverage: covered versus not covered; participation in labor force: yes versus no; parity: nil, 1–3, and 4+; source of drinking water: improved versus unimproved; sanitation: improved versus unimproved; ever had a terminated pregnancy: yes versus no; body mass index: underweight, normal, overweight, and obese; ever used anything or tried to delay or avoid getting pregnant: yes versus no; anemia status: anemic versus not anemic; smoking/use tobacco product: yes versus no; alcohol use: yes versus no.

The wealth indicator weights were determined by DHS using the principal component analysis (PCA) technique to assign the wealth indicator weights. Wealth indicator variable scores were allocated and standardized using household assets such as wall type, floor type, roof type, water supply, sanitation facilities, radio, electricity, television, refrigerator, cooking fuel, furniture, and the number of persons per room. The factor loadings and z-scores have then been determined. The indicator values were multiplied by the factor loadings for each household and summarized to generate the wealth index value of the household. To categorize the overall scores into wealth quintiles, the standardized z-score was used: poorest, poorer, middle, richest, and richest [24]. The factors examined in this study are based on previous studies related to renal failure [11, 12, 20, 21].

#### 2.2.3. Ethical Consideration

BDHS data is publicly available. We sought permission from MEASURE DHS/ICF International, and access to the data was granted after our intent for the request was assessed and approved. MEASURE DHS Program is consistent with the standards for ensuring the protection of respondents' privacy. ICF International ensures that the survey complies with the U.S. Department of Health and Human Services regulations for the respect of human subjects. No further approval was required as secondary data analysis was conducted in this study. More details about data and ethical standards are available at http://goo.gl/ny8T6X.

#### 2.2.4. Analytical Approach

The survey (‘svy') module was used to adjust for survey design. A variance inflation factor of 10 was used to determine multicollinearity known to cause major concerns in regression models [25, 26]. However, no variable was excluded from the model as they were not found to be interdependent. Percentage and multivariable logistic regression model were used to estimate the prevalence of renal failure and its associated factor, respectively [27]. The statistical signiﬁcance was determined at *p* < 0.05. Stata Version 14 (StataCorp., College Station, TX, USA) was used for data analysis.

## 3. Results

Table 1 showed the sample size for women who have suffered renal failure and those who have not suffered from renal failure across selected characteristics. The prevalence of renal failure increased by increasing age of women and was the highest among women aged 45–49 years (11.8%). Among rural dwellers, Bururi, Makamba, and Rumonge geographical regions were 5.4%, 10.3%, 21.2%, and 22.0%, respectively. Women who had no formal education reported a renal failure prevalence of 6.9%. The richer, richest, ever married, or smoked women had about 6.0% prevalence of renal failure. Women who had given birth to at least 4 children or ever had a terminated pregnancy had an 8.3% and 9.4% prevalence of renal failure, respectively. Overall, the prevalence of renal failure was 5.0% (95% CI: 4.4%, 5.7%) among Burundi women.

In Table 2, marginal effect analysis was conducted to decipher the effects of the factors associated with renal failure. From the predictive marginal effects results, assuming that the distribution of all factors remained the same among women, but every woman was aged 45–49 years, we would expect 10.2% of renal failure. If every woman had a resident in a rural area, we would expect 5.5% of renal failure. If, instead, the distribution of women's age, residence, and geographical region was as observed and other covariates remained the same among women, but no woman had a formal education, we would expect about 5.5% of renal failure. Furthermore, if, instead, the spread of the aforementioned variables was as observed and other covariates remained equal among women, but every woman had a history of a terminated pregnancy, we would expect 6.7% of renal failure. In Table 2, we obtained the predictive marginal effects of the factors associated with renal failure.

Results from Table 3 showed that higher-aged women were more likely to have a renal failure when compared with women aged 15–19 years. Rural dwellers were 1.65 times as likely to have a renal failure when compared with women in the urban residence (AOR = 1.65; 95% CI: 1.24, 2.20). Women who had secondary + education had a 39% reduction in the odds of renal failure when compared with women with no formal education (AOR = 0.61; 95% CI: 0.46, 0.81). Health insurance coverage accounted for a 23% reduction in the odds of renal failure when covered with women who were not covered by health insurance (AOR = 0.77; 95% CI: 0.63, 0.93). Women who had a terminated pregnancy were 1.50 times as likely to have a renal failure when compared with women with no history of terminated pregnancy (AOR = 1.50; 95% CI: 1.24, 1.82). Furthermore, women who ever used anything or tried to delay or avoid getting pregnant were 1.32 times as likely to have a renal failure when compared with women without a history of using anything to delay getting pregnant.

## 4. Discussion

This study has become the first in Burundi to examine the prevalence of renal failure and associated factors among women of reproductive age. The overall prevalence was approximately 5%, which is less than 7% to 15% in Eastern Africa countries with a pooled prevalence of about 11% [28]. In another study conducted in rural Eastern Africa countries, the estimated chronic renal failure prevalence was 6.8% [21]. However, there were variations in prevalence by region, being 12.5% in eastern Uganda, 3.9% in southwestern Uganda, and 3.7% in western Kenya [21]. In a systematic review involving the general population across all ages and gender, there was a pooled prevalence of 4.6% for chronic renal failure stages 3–5 in the African continent which is similar to the prevalence in our study [18]. Moreover, the prevalence of renal failure among Burundi women (5.0%) is less than the overall prevalence of renal failure in sub-Saharan Africa (13·9%) [11]. In Africa, renal failure is a public health problem, mainly attributed to high-risk conditions [18].

We found multiple results on renal failure consistent with previous literature. First, we found that a history of contraceptive use was a risk factor for renal failure. Compared with women who never used contraceptive methods, those who ever used contraceptive had higher odds of renal failure. The findings from our study are consistent with previous reports regarding the effects of hormonal contraceptives on renal failure, as various epidemiological studies have shown an association between hormonal contraceptive use and renal failure (urinary albumin loss) [29–33]. From a previous study, the start of hormonal contraceptive was independently associated with worsening of renal function, while stopping hormonal contraceptive use resulted in an improvement [29]. The results suggested that long-term hormonal contraceptive use was deleterious from the cardiovascular and renal point of view. Although hormonal contraceptives have been used for decades, not much attention has been drawn to the renal adverse events associated with these agents. Epidemiological and pathophysiological data on hormonal contraceptive use and the renal outcome, e.g., albuminuria and renal function, are limited [29, 34]. Interestingly, some studies have recently described an association between the use of hormonal contraceptives and albuminuria [30, 32]. Higher levels of albuminuria are considered an early marker of vascular endothelial damage and are related to an increased risk of progressive renal failure and excess cardiovascular morbidity and mortality [29, 34]. Our study has corroborated to suggest that contraceptive use predisposes women to renal failure in Burundi.

It is of interest that our study was able to establish an age-related association with renal failure. It is well known that renal function increases with older age. This is consistent with the results of previous studies [21, 35]. In a previous study, the odds ratios of chronic renal failure increased by every 10-year increase in age among subjects older than 30 years [35]. In addition, the rural dwellers had higher odds of renal failure. Conversely, education and health insurance coverage were protective factors of renal failure among women of reproductive age in Burundi. Improved educational attainment is known to enhance accessibility to health information and good healthcare-seeking behavior. In a previous study, those with less than high school education were more likely to have renal failure, when compared with those with college education [36]. Moreover, health insurance coverage could be a channel for utilizing health care services including regular check-ups as well as early detection and treatment of health problems. Furthermore, we found an association between renal failure and geographical region. In previous studies which involved geospatial analysis, there was significant heterogeneous distribution for all impaired kidney function conditions [2, 21].

Furthermore, pregnancy termination was associated with higher odds of renal failure. Pregnancy termination occurs either spontaneously as miscarriage or induced as abortion. It may be safe or results in complications and death [37]. Substantial blood loss and infection can result in acute renal failure. Both of these can occur during unsafe pregnancy termination. In a previous study, major blood loss and sepsis played a role in the precipitation of renal ischemia [38]. Another study reported a case of nonoliguric acute renal failure and abortion following the ingestion of an overdose of metamizol in an otherwise healthy girl [39]. The adverse effects in the use and/or dose of over-the-counter medicine especially in the advent of unwanted pregnancy termination are common in many resource-constrained settings with poor abortion care services, which can make the body organs fail. The prohibition of abortion in Burundi could also promote the use of nonprescription medicine and consequently lead to organ failure [40]. Abortion in Burundi is only legal if the abortion will save the woman's life or if the pregnancy gravely endangers the woman's physical or potentially mental health [41]. In Burundi, two certified physicians must agree that the pregnancy is threatening before giving medical assistance and that could be another reason why women may opt for abortion services in less formal facilities.

### 4.1. Strengths and Limitations

Population-based data was analyzed in this study, making the findings generalizable to women of reproductive age in Burundi. Nonetheless, only an association was established and not causality due to the cross-sectional nature of the data. Also, we were unable to explore other contributory risk factors, such as salt intake, psychosocial stress, and other endogenous factors. Since we utilized secondary data, we could not determine whether respondents had acute or chronic renal failure as the outcome variable was based on women's self-report. Reporting renal failure could be limiting as women's awareness and knowledge about the disease would determine their report.

## 5. Conclusion

The prevalence of renal failure among women of reproductive age in Burundi is an issue of concern. The risk factors for renal failure identified in this study will be useful in the design of the intervention and foster the implementation of screening, especially among the most-at-risk populations to ensure early detection and prevention and initiate treatment of modifiable risk factors. Efforts should be made to develop and implement social and behavior change communication strategies that will target key populations and spur them to screen for renal disease. Strategies should be designed to screen for and manage high-risk conditions such as hypertension and diabetes mellitus in an effort to decrease the incidence of renal failure. In areas where there are insufficient numbers of physicians and nurses, other allied health workers could be trained to screen for this condition at a local level, with clearly defined criteria and referral system. Patients with renal disease should be referred to a nephrologist at an early stage so as to institute measures to retard progression and plan timely transplantation and/or dialysis; this is particularly important where related donors may be available as a cost-effective strategy.

## Figures and Tables

**Table 1:** Sociodemographic characteristics of respondents and the distribution of renal failure (*n* = 17,269).

Variable	Number of respondents (%)	Renal failure		*P*
		Yes (%)	No (%)	
Age				<0.001^*∗*^
15–19	3968 (23.0)	83 (2.1)	3885 (97.9)	
20–24	3250 (18.8)	93 (2.9)	3157 (97.1)	
25–29	2936 (17.0)	122 (4.2)	2814 (95.8)	
30–34	2430 (14.1)	135 (5.6)	2295 (94.4)	
35–39	1941 (11.2)	127 (6.5)	1814 (93.5)	
40–44	1541 (8.9)	138 (9.0)	1403 (91.0)	
45–49	1203 (7.0)	142 (11.8)	1061 (88.2)	

Residential status				<0.001^*∗*^
Urban	3671 (21.3)	105 (2.9)	3566 (97.1)	
Rural	13598 (78.7)	735 (5.4)	12863 (94.6)	

Geographical region				<0.001^*∗*^
Bubanza	817 (4.7)	20 (2.5)	797 (97.5)	
Bujumbura Rural	950 (5.5)	28 (3.0)	922 (97.0)	
Bururi	933 (5.4)	96 (10.3)	837 (89.7)	
Cankuzo	856 (5.0)	5 (0.6)	851 (99.4)	
Cibitoke	922 (5.3)	28 (3.0)	894 (97.0)	
Gitega	1153 (6.7)	62 (5.4)	1091 (94.6)	
Karusi	933 (5.4)	6 (0.6)	927 (99.4)	
Kayanza	1084 (6.3)	47 (4.3)	1037 (95.7)	
Kirundo	982 (5.7)	26 (2.6)	956 (97.4)	
Makamba	941 (5.5)	199 (21.2)	742 (78.8)	
Muramvya	913 (5.3)	5 (0.6)	908 (99.4)	
Muyinga	996 (5.8)	5 (0.6)	991 (99.5)	
Mwaro	886 (5.1)	19 (2.1)	867 (97.9)	
Ngozi	1032 (6.0)	20 (1.9)	1012 (98.1)	
Rutana	904 (5.2)	42 (4.7)	862 (95.3)	
Ruyigi	908 (5.3)	0 (0.0)	908 (100.0)	
Bujumbura Mairie	1155 (6.7)	33 (2.9)	1122 (97.1)	
Rumonge	904 (5.2)	199 (22.0)	705 (78.0)	

Education				<0.001^*∗*^
No formal education	5879 (34.0)	405 (6.9)	5474 (93.1)	
Primary	6555 (38.0)	312 (4.8)	6243 (95.2)	
Secondary+	4835 (28.0)	123 (2.5)	4712 (97.5)	

Religion				0.078
Christianity	16285 (94.3)	806 (5.0)	15479 (95.0)	
Islam	681 (3.9)	26 (3.8)	655 (96.2)	
Traditional/no religion	303 (1.8)	8 (2.6)	295 (97.4)	

Exposure to media				0.259
Yes	9109 (52.8)	459 (5.0)	8650 (95.0)	
No	8160 (47.2)	381 (4.7)	7779 (95.3)	

Wealth quintiles				<0.001^*∗*^
Poorest	3333 (19.3)	106 (3.2)	3227 (96.8)	
Poorer	3393 (19.7)	150 (4.4)	3243 (95.6)	
Middle	3560 (20.6)	163 (4.6)	3397 (95.4)	
Richer	3638 (21.1)	220 (6.1)	3418 (93.9)	
Richest	3345 (19.4)	201 (6.0)	3144 (94.0)	

Marital status				<0.001^*∗*^
Unmarried	6280 (36.4)	156 (2.5)	6124 (97.5)	
Currently married/living with a partner	9559 (55.4)	592 (6.2)	8967 (93.8)	
Formerly married	1430 (8.3)	92 (6.4)	1338 (93.6)	

Health insurance coverage				0.013^*∗*^
Covered	4003 (23.2)	165 (4.1)	3838 (95.9)	
Not covered	13266 (76.8)	675 (5.1)	12591 (94.9)	

Participation in labor force				<0.001^*∗*^
Yes	13311 (77.1)	733 (5.5)	12578 (94.5)	
No	3958 (22.9)	107 (2.7)	3851 (97.3)	

Parity				<0.001^*∗*^
Nil	6188 (35.8)	151 (2.4)	6037 (97.6)	
1–3	5267 (30.5)	209 (4.0)	5058 (96.0)	
4+	5814 (33.7)	480 (8.3)	5334 (91.7)	

Source of drinking water				<0.001^*∗*^
Improved	14358 (83.1)	660 (4.6)	13698 (95.4)	
Unimproved	2911 (16.9)	180 (6.2)	2731 (93.8)	

Sanitation				0.002^*∗*^
Improved	9944 (57.6)	528 (5.3)	9416 (94.7)	
Unimproved	7325 (42.4)	312 (4.3)	7013 (95.7)	

Ever had a terminated pregnancy				<0.001^*∗*^
Yes	2110 (12.2)	199 (9.4)	1911 (90.4)	
No	15159 (87.8)	641 (4.2)	14518 (95.8)	

Source of drinking water				<0.001^*∗*^
Improved	14358 (83.1)	660 (4.6)	13698 (95.4)	
Unimproved	2911 (16.9)	180 (6.2)	2731 (93.8)	

Sanitation				0.002^*∗*^
Improved	9944 (57.6)	528 (5.3)	9416 (94.7)	
Unimproved	7325 (42.4)	312 (4.3)	7013 (95.7)	

Ever had a terminated pregnancy				<0.001^*∗*^
Yes	2110 (12.2)	199 (9.4)	1911 (90.4)	
No	15159 (87.8)	641 (4.2)	14518 (95.8)	

Body mass index				0.236
Underweight	1509 (17.6)	59 (3.9)	1450 (96.1)	
Normal	6258 (72.9)	289 (4.6)	5969 (95.4)	
Overweight	630 (7.3)	37 (5.9)	593 (94.1)	
Obese	184 (2.1)	7 (3.8)	177 (96.2)	

Ever used anything or tried to delay or avoid getting pregnant				0.001^*∗*^
Yes	5835 (33.8)	328 (5.6)	5507 (94.4)	
No	11434 (66.2)	512 (4.5)	10922 (95.5)	

Anemia status				0.643
Anemic	3176 (37.2)	149 (4.7)	3027 (95.3)	
Not anemic	5363 (62.8)	240 (4.5)	5123 (95.5)	

Smoking/use tobacco product				0.056
Yes	849 (4.9)	53 (6.2)	796 (93.8)	
No	16420 (95.1)	787 (4.8)	15633 (95.2)	

Alcohol use				<0.001^*∗*^
Yes	8145 (47.2)	341 (4.2)	7804 (95.8)	
No	9124 (52.8)	499 (5.5)	8625 (94.5)	

^*∗*^Significant at *p* < 0.05.

**Table 2:** Marginal predictive model of the factors associated with renal failure.

Variable	Marginal effect (%)	95% CI	*P*
Age			
15–19	2.8	2.0, 3.5	<0.001^*∗*^
20–24	3.5	2.8, 4.2	<0.001^*∗*^
25–29	4.5	3.7, 5.3	<0.001^*∗*^
30–34	5.4	4.5, 6.3	<0.001^*∗*^
35–39	6.0	5.0, 7.0	<0.001^*∗*^
40–44	7.5	6.2, 8.7	<0.001^*∗*^
45–49	10.2	8.5, 11.8	<0.001^*∗*^

Residential status			
Urban	3.6	2.8, 4.4	<0.001^*∗*^
Rural	5.5	5.1, 5.9	<0.001^*∗*^

Geographical region			
Bubanza	2.3	1.3, 3.3	<0.001^*∗*^
Bujumbura Rural	3.1	2.0, 4.2	<0.001^*∗*^
Bururi	11.2	9.1, 13.3	<0.001^*∗*^
Cankuzo	0.5	0.1, 1.0	0.025^*∗*^
Cibitoke	3.0	1.9, 4.1	<0.001^*∗*^
Gitega	5.2	3.9, 6.5	<0.001^*∗*^
Karusi	0.6	0.1, 1.1	0.014^*∗*^
Kayanza	4.2	3.0, 5.4	<0.001^*∗*^
Kirundo	2.3	1.4, 3.2	<0.001^*∗*^
Makamba	20.8	18.1, 23.4	<0.001^*∗*^
Muramvya	0.5	0.1, 1.0	0.025^*∗*^
Muyinga	0.5	0.1, 0.9	0.025^*∗*^
Mwaro	2.1	1.2, 3.1	<0.001^*∗*^
Ngozi	1.8	1.0, 2.6	<0.001^*∗*^
Rutana	4.4	3.0, 5.7	<0.001^*∗*^
Ruyigi		—	—
Bujumbura Mairie	5.9	3.6, 8.2	<0.001^*∗*^
Rumonge	21.5	18.8, 24.3	<0.001^*∗*^

Education			
No formal education	5.6	5.0, 6.2	<0.001^*∗*^
Primary	5.5	4.9, 6.0	<0.001^*∗*^
Secondary+	3.7	3.0, 4.4	<0.001^*∗*^

Wealth quintiles			
Poorest	4.7	3.9, 5.6	<0.001^*∗*^
Poorer	5.4	4.6, 6.2	<0.001^*∗*^
Middle	4.9	4.2, 5.6	<0.001^*∗*^
Richer	5.4	4.7, 6.1	<0.001^*∗*^
Richest	5.1	4.4, 5.9	<0.001^*∗*^

Marital status			
Unmarried	4.9	3.9, 5.8	<0.001^*∗*^
Currently married/living with a partner	5.2	4.8, 5.7	<0.001^*∗*^
Formerly married	5.0	4.0, 6.0	<0.001^*∗*^

Health insurance coverage			
Covered	4.3	3.7, 4.9	<0.001^*∗*^
Not covered	5.4	5.0, 5.8	<0.001^*∗*^

Participation in labor force			
Yes	5.3	4.9, 5.6	<0.001^*∗*^
No	4.5	3.7, 5.3	<0.001^*∗*^

Source of drinking water			
Improved	5.0	4.7, 5.4	<0.001^*∗*^
Unimproved	5.7	4.9, 6.5	<0.001^*∗*^

Sanitation			
Improved	5.1	4.7, 5.6	<0.001^*∗*^
Unimproved	5.1	4.6, 5.7	<0.001^*∗*^

Ever had a terminated pregnancy			
Yes	6.7	5.8, 7.6	<0.001∗
No	4.8	4.5, 5.1	<0.001∗

Ever used anything or tried to delay or avoid getting pregnant			
Yes	5.9	5.3, 6.5	<0.001^*∗*^
No	4.7	4.3, 5.1	<0.001^*∗*^

Alcohol use			
Yes	5.2	4.6, 5.7	<0.001^*∗*^
No	5.1	4.7, 5.5	<0.001^*∗*^

^*∗*^Significant at *p* < 0.05; CI: confidence interval; na: not estimated due to lack of data.

**Table 3:** Multivariable logistic regression of the factors associated with renal failure.

Variable	Adjusted odds ratio	95% CI	*P*
Age			
15–19	1.00		
20–24	1.32	0.94, 1.86	0.109
25–29	1.73	1.20, 2.50	0.003^*∗*^
30–34	2.15	1.46, 3.15	<0.001^*∗*^
35–39	2.43	1.65, 3.59	<0.001^*∗*^
40–44	3.18	2.14, 4.73	<0.001^*∗*^
45–49	4.74	3.16, 7.11	<0.001^*∗*^

Residential status			
Urban	1.00	1.24, 2.20	0.001^*∗*^
Rural	1.65		

Geographical region			
Bubanza	1.00		
Bujumbura Rural	1.36	0.75, 2.45	0.306
Bururi	5.67	3.42, 9.38	<0.001^*∗*^
Cankuzo	0.23	0.09, 0.62	0.004^*∗*^
Cibitoke	1.33	0.74, 2.39	0.343
Gitega	2.38	1.41, 4.02	0.001^*∗*^
Karusi	0.25	0.10, 0.64	0.004^*∗*^
Kayanza	1.91	1.10, 3.29	0.020^*∗*^
Kirundo	1.00	0.55, 1.82	0.995
Makamba	12.50	7.72, 20.23	<0.001^*∗*^
Muramvya	0.22	0.08, 0.60	0.003^*∗*^
Muyinga	0.20	0.07, 0.53	0.001^*∗*^
Mwaro	0.93	0.49, 1.77	0.824
Ngozi	0.78	0.41, 1.47	0.443
Rutana	1.96	1.13, 3.41	0.017^*∗*^
Ruyigi	Na	—	—
Bujumbura Mairie	2.72	1.47, 5.06	0.001^*∗*^
Rumonge	13.12	1.13, 3.41	<0.001^*∗*^

Education			
No formal education	1.00		
Primary	0.97	0.81, 1.16	0.756
Secondary+	0.61	0.46, 0.81	0.001^*∗*^

Wealth quintiles			
Poorest	1.00		
Poorer	1.16	0.88, 1.53	0.282
Middle	1.03	078, 1.37	0.812
Richer	1.17	0.88, 1.54	0.272
Richest	1.10	0.82, 1.49	0.524

Marital status			
Unmarried	1.00		
Currently married/living with a partner	1.09	0.81, 1.45	0.580
Formerly married	1.02	0.71, 1.46	0.918

Health insurance coverage			
Covered	0.77	0.63, 0.93	0.008^*∗*^
Not covered	1.00		

Participation in labor force			
Yes	1.20	0.94, 1.52	0.140
No	1.00		

Source of drinking water			
Improved	0.86	0.71, 1.04	0.126
Unimproved	1.00		

Sanitation			
Improved	0.99	0.84, 1.19	0.986
Unimproved	1.00		

Ever had a terminated pregnancy			
Yes	1.50	1.24, 1.82	<0.001^*∗*^
No	1.00		

Ever used anything or tried to delay or avoid getting pregnant			
Yes	1.32	1.11, 1.57	0.001^*∗*^
No	1.00		

Alcohol use			
Yes	1.01	0.85, 1.19	0.916
No	1.00		

^*∗*^Significant at *p* < 0.05; AOR of 1.00 indicates the reference category; CI: confidence interval; na: not estimated due to lack of data.

## Data Availability

Data for this study were sourced from Demographic and Health Surveys (DHS) and available at http://dhsprogram.com/data/available-datasets.cfm.
